# A Novel *SPAST* Variant Associated with Isolated Spastic Paraplegia

**DOI:** 10.1155/2023/4553365

**Published:** 2023-12-31

**Authors:** Helle Høyer, Ola Nakken, Trygve Holmøy

**Affiliations:** ^1^Department of Medical Genetics, Telemark Hospital, Skien, Norway; ^2^Department of Neurology, Akershus University Hospital, Lørenskog, Norway; ^3^Institute of Clinical Medicine, University of Oslo, Oslo, Norway

## Abstract

Genetic variants in *SPAST* are the most common cause of hereditary spastic paraplegia (HSP), entitled spastic paraplegia type 4 (SPG4). Inheritance is autosomal dominant, and age of onset can vary from childhood to adulthood. Pathogenic *SPAST* variants are often observed in isolated cases, likely due to reduced penetrance and clinical variability. We report an isolated case of SPG4 associated with a novel likely pathogenic variant in *SPAST*. A 38-year-old woman presented with an eight-year history of progressive difficulty walking. Neurological examination revealed spastic paraparesis in the absence of upper motor neuron dysfunction, sensory deficits, or intellectual disability. Magnetic resonance imaging (MRI) of the brain and spinal cord was normal. No family members had similar complaints. Genetic analysis revealed a novel heterozygous sequence variant in *SPAST*, c.1751A > G p.(Asp584Gly) (NM_014946.4). The affected amino acid is highly conserved among orthologue and paralogue species. Four other nucleotide substitutions predicted to affect the same amino acid, a “hot spot”, have been reported previously in adult-onset HSP. This report describes a novel *SPAST* variant in a female with HSP without a known family history of the disorder.

## 1. Introduction

Hereditary spastic paraplegia (HSP) consists of a group of neurological disorders characterised by length-dependent corticospinal tract and dorsal column degeneration, typically resulting in bilateral lower limb spasticity, hyperreflexia, and extensor plantar responses [1]. In a population-based study in Norway, the overall prevalence of HSP was 7.4/100,000. The prevalence of cases considered to be autosomal dominant or autosomal recessive based on family history was 5.5/100, 000 and 0.6/100,000, respectively. The prevalence of isolated cases, i.e., cases without a known family history, was 1.3/100 000 [2]. A German study has more recently reported that 40% of cases are isolated [3].

Pathogenic and likely pathogenic variants in over 80 genes can cause spastic paraplegia [4]. Several hundred variants in *SPAST* can underlie spastic paraplegia type 4 (SPG4). Pathogenic variants in *SPAST* account for approximately 25% of autosomal dominant HSP, and the *SPAST* gene is the most frequently mutated gene in isolated cases [2, 5]. Wide inter- and intrafamilial clinical variability exists, and the age of onset ranges from the first to the eighth decade. Penetrance is almost complete after the seventh decade [6]. We report an isolated case with a novel likely pathogenic variant in *SPAST*.

## 2. Case Presentation

The patient is a 38-year-old woman who works full-time as a secretary. She underwent bilateral femoral osteotomies at age ten years and developed low back pain without sciatica from age 18. Magnetic resonance imaging (MRI) revealed spondylolisthesis at L4/5 with bilateral compression of L5 nerve roots. At age 36 years, she was referred to a neurologist with a six-year history of difficulty walking in the absence of other complaints. She was able to walk approximately 100 meters without assistance. Her gait disturbance was least pronounced in the morning and after rest. She had no upper limb, visual, neuropsychiatric, or urinary symptoms.

Neurological examination was normal except for spastic paraparesis with bilateral ankle clonus, patellar subclonus, and positive Babinski sign. She did not have nystagmus, dysarthria, dysphagia, muscle wasting, extrapyramidal signs, or sensory loss. Her HSP rating scale (HSPRS) score was 17 of 52 [7]. She had low levels of serum vitamin B9 (4 nmol/l) and D (29 nmol/l), but there was no evidence of metabolic or inflammatory disease. MRI of the brain and the spinal cord was normal.

Introduction of baclofen 25 mg three times a day had some effect on her spasticity. However, her HSPRS score remained unchanged at 18-month follow-up.

**Figure 1:**
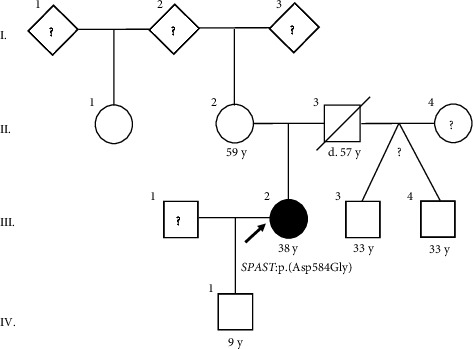
Pedigree. Arrow = proband, black symbol = affected by SPG4, white symbols = not reported by proband to have difficulty walking, and white symbols with ? = unknown status. d = died, y = years.

Her mother who is 59 years old and her mother's half-sister have no difficulty walking, according to the proband. Her father died at the age of 57 years; he had low back pain and no difficulty walking. Our patient reports that her two 33-year-old half-siblings (shared father) and her 12-year-old son have no difficulty walking. The pedigree is shown in Figure 1. It has not been possible to interview, examine, or collect blood samples from the patient's relatives.

**Figure 2:**
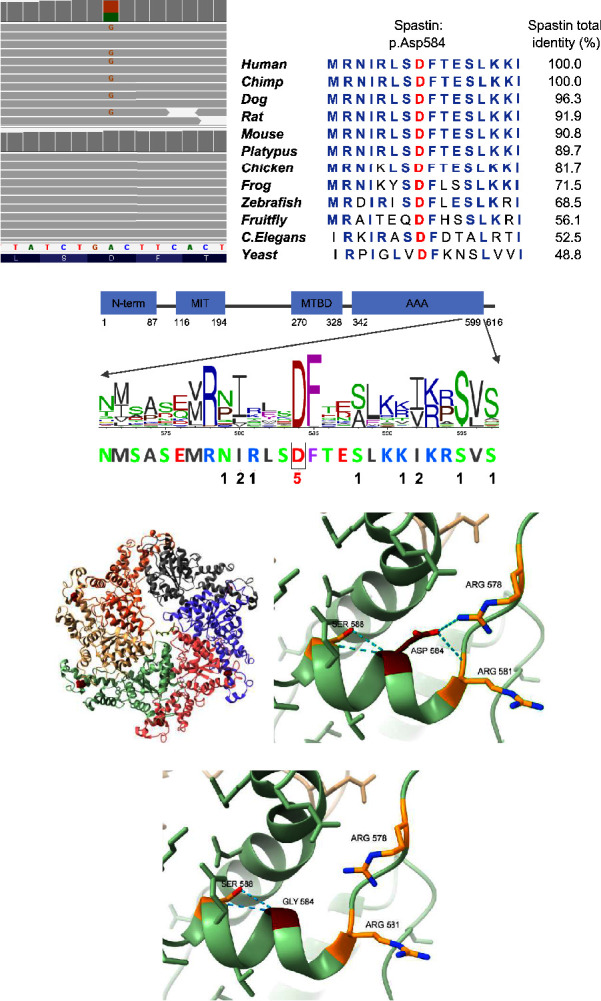
Sequencing data, conservation, and *in silico* modelling. (a) Sequencing data shown in integrative genomics viewer (Broad Institute) for the proband (upper panel) and a control (lower panel). The nucleotide substitution from adenine (A) to guanine (G) is shown in orange. (b) Evolutionary conservation of the amino acid affected by the spastin p.(Asp584Gly) variant across different species. The affected amino acid is shown in red. Blue indicates amino acids that are similar to the human sequence. Data from Alamut Visual Software (Sophia Genetics) and protein BLAST (https://blast.ncbi.nlm.nih.gov/Blast.cgi). (c) Spastin domain structure and paralog conservation of amino acids 571–597 among 14 related orthologues identified from the protein data bank (PDB). Alignment was created by VarSite (EMBL-EBI). The amino acids in the paralogue alignment are coloured by residue type. Shown below the alignment is the number of disease-causing variants at each position based on the p.(Asp584Gly) substitution reported in our case and reports in the human gene mutation database (HGMD) professional version 2022.2 (QiaGen). N-term = N-terminal sequence, MIT = microtubule interacting and trafficking domain, MTBD = microtubule-binding domain, and AAA = ATPase associated with various cellular activities. The asparagine (D) residue at position 584 is marked with a box. (d) Cartoon modelling of the human spastin hexamonomer. There is one colour for each monomer. The Asp584 residue is shown in red in each of the six monomers. (e) Localization of the p.Asp584 residue (dark red) and its interacting amino acids (orange). Hydrogen bonds (light blue) predicted with p.Arg578, p.Arg581, and p.Ser588. (f) Localization of the p.(Asp584Gly) substitution (dark red) and interacting amino acids (orange). Hydrogen bonds (light blue) are predicted to be formed with p.Ser588, but not with p.Arg578 and p.Arg581. The position of the new amino acid change within the structure was estimated using the Rotamers tool with standard parameters. The modelling in (d), (e), and (f) was performed using ChimeraX-1.5 on the spastin (PDB ID: 6PEN) crystal structure.

Genetic analyses of the proband included multiplex ligation-dependent probe amplification (MLPA) of *SPAST, REEP1, ATL1, SPG11*, and *SPG7* and next-generation sequencing (NGS) of 115 genes associated with HSP. The NGS panel was based on Genomics England PanelApp (https://panelapp.genomicsengland.co.uk/) and included red and amber genes from the following panels: hereditary spastic paraplegia (version 1.276), hereditary spastic paraplegia—adult onset (version 1.88), and hereditary spastic paraplegia—childhood onset (version 2.121). NGS revealed a heterozygous variant in *SPAST*, c.1751A > G p.(Asp584Gly) (NM_014946.4) (Figure 2(a)). This variant has not previously been reported, but four other nucleotide substitutions affecting the same codon p.(Asp584Asn), p.(Asp584Glu), p.(Asp584His), and p.(Asp584Val) have been reported to cause HSP [6, 8–10]. The p.(Asp584Gly) variant is absent from large population databases, i.e., more than 250 000 *SPAST* alleles annotated in GnomAD and 13 000 alleles annotated in esp6500. The p.(Asp584Gly) substitution targets an evolutionarily conserved residue in orthologues (Figure 2(b)) and paralogues (Figure 2(c)). The variant is localized to the AAA cassette domain (Figure 2(c)), a hot spot for pathogenic variation [6]. *In silico* modelling of the spastin homohexamer, based on the spastin (Protein Data Bank (PDB) ID: 6PEN) crystal structure using ChimeraX-1.5, showed that the p.(Asp584Gly) is situated in the outer part of the protein (Figure 2(d)). The substitution from aspartic acid to glycine results in a side chain alteration from a negative charge to a single hydrogen atom predicted to disrupt hydrogen bonds with Arg578 and Arg581, potentially resulting in structural and/or functional effects (Figures 2(e), 2(f)).

Based on the ACMG (American College of Medical Genetics) criteria PM1, PM2, PM5, and PP3 [11], c.1751A > G is likely pathogenic.

## 3. Discussion/Conclusions

SPG4 is caused by both missense and loss of function variants altering *SPAST* and is typically associated with slowly progressive lower extremity weakness and corticospinal tract signs, with or without sphincter disturbances and deep sensory loss, but without other neurological symptoms or signs [12]. This is in keeping with the phenotype in our patient.

The p.(Asp584Gly) variant we identified has not been reported previously. However, four other missense variants affecting the same asparagine residue are known to cause HSP [6, 8–10]. Conservation is complete across orthologue species and paralogue proteins (Figures 2(b), 2(c)), suggesting that asparagine in position 584 is important for normal spastin function.

Spastin has a key role in neuronal axons where it modulates microtubule number, length, and motility [13]. Parodi et al. analysed a cohort of 842 patients with SPG4 to assess genotype-phenotype correlations. Age of onset in individuals carrying missense variants tended to be in the first to second decade, whereas individuals with loss of function variants often experienced symptoms starting in the second to sixth decade. There was no difference in clinical severity, but intellectual disability was more common in individuals with missense variants [6]. Our patient who carried a missense variant had the onset in her early thirties and no intellectual disability. Interestingly, other patients carrying missense variants affecting the same codon also have relatively late onset ranging from 21 to 40 years [6, 8–10]. An individual heterozygous for p.(Asp584His) had not developed symptoms at the age of 70 [10]. Variation affecting p.Asp584 may predispose to a phenotype often seen in patients with loss of function variants. The patient presented in our study reported that no other family members had symptoms similar to hers. However, since it was not possible to confirm her negative family history, other family members may carry the variant. Family history for SPG4 may appear to be negative due to age-related/reduced penetrance or death prior to the onset of the disorder [4]. The proportion of cases caused by a *de novo* variant is low [4].

In conclusion, we report a novel heterozygous likely pathogenic missense variant in *SPAST* in an apparently isolated case of pure HSP.

## Data Availability

The data used and analysed during the current study are available from the corresponding author upon reasonable request.
